# Brain Lateralization in Mice Is Associated with Zinc Signaling and Altered in Prenatal Zinc Deficient Mice That Display Features of Autism Spectrum Disorder

**DOI:** 10.3389/fnmol.2017.00450

**Published:** 2018-01-15

**Authors:** Stefanie Grabrucker, Jasmin C. Haderspeck, Ann Katrin Sauer, Nadine Kittelberger, Harun Asoglu, Alireza Abaei, Volker Rasche, Michael Schön, Tobias M. Boeckers, Andreas M. Grabrucker

**Affiliations:** ^1^Institute for Anatomy and Cell Biology, Ulm University, Ulm, Germany; ^2^Cellular Neurobiology and Neuro-Nanotechnology Laboratory, Department of Biological Sciences, University of Limerick, Limerick, Ireland; ^3^WG Molecular Analysis of Synaptopathies, Neurology Department, Neurocenter of Ulm University, Ulm, Germany; ^4^Core Facility Small Animal Imaging, Ulm University, Ulm, Germany; ^5^Department of Internal Medicine II, Ulm University Medical Center, Ulm, Germany; ^6^Bernal Institute, University of Limerick, Limerick, Ireland; ^7^Health Research Institute (HRI), University of Limerick, Limerick, Ireland

**Keywords:** Zn, hemisphere dominance, ASD, synapse, trace metal, connectivity

## Abstract

A number of studies have reported changes in the hemispheric dominance in autism spectrum disorder (ASD) patients on functional, biochemical, and morphological level. Since asymmetry of the brain is also found in many vertebrates, we analyzed whether prenatal zinc deficient (PZD) mice, a mouse model with ASD like behavior, show alterations regarding brain lateralization on molecular and behavioral level. Our results show that hemisphere-specific expression of marker genes is abolished in PZD mice on mRNA and protein level. Using magnetic resonance imaging, we found an increased striatal volume in PZD mice with no change in total brain volume. Moreover, behavioral patterns associated with striatal lateralization are altered and the lateralized expression of dopamine receptor 1 (DR1) in the striatum of PZD mice was changed. We conclude that zinc signaling during brain development has a critical role in the establishment of brain lateralization in mice.

## Introduction

Although the right and left brain hemisphere appear as mirror image on first impression, there are a large number of publications that indicate fundamental differences in the processing of information between the hemispheres. The dominance of one hemisphere for a specific brain function (for example, right hemisphere dominance for spatial learning, or left hemisphere dominance for the detection of species-specific communication calls) is often conserved across various taxa of vertebrates (birds, reptiles, fish, and mammals). There, asymmetries in the perception process can be observed, for example, on behavioral level (Vallortigara and Rogers, 2005), but also on the molecular level. However, the underlying neural mechanisms of the cerebral lateralization are not yet fully known (Vallortigara and Rogers, 2005).

Previous studies have shown an impairment of hemispheric dominance with interference of acoustic perception, speech perception, and motor functions in autistic patients (Hollander et al., 2005; Haznedar et al., 2006; Whitehouse and Bishop, 2008; Jones et al., 2009; Bonnel et al., 2010; Hitoglou et al., 2010; Wan et al., 2012; Floris et al., 2013; Lindell and Hudry, 2013). For example, asymmetry reversal, i.e., reduced asymmetry patterns in different areas of the cortex associated with language has been described in autistic patients (Herbert et al., 2002, 2005; Rojas et al., 2002; Boddaert et al., 2003; De Fossé et al., 2004). Intriguingly, impairments in speech and communication are among the core features of autism spectrum disorder (ASD). Moreover, regions involved in visual face processing have been shown to display abnormal asymmetry, which might explain deficits in social interactions in patients (Herbert et al., 2002). During motor imitation tasks, significantly higher activation of the right hemisphere could be observed in individuals with autism (Dawson, 1983).

In our previous studies, we were able to create a mouse model that displays ASD like features by induction of prenatal zinc deficiency (Grabrucker et al., 2014, 2016). Prenatal zinc deficiency has been associated with ASD in humans (Sayehmiri et al., 2015; Arora et al., 2017). In line with this, prenatal zinc deficient (PZD) mice display ASD like behavioral impairments that are present in adulthood although zinc deficiency was only induced during brain development. For example, prenatal zinc deficiency in mice induces impairments in vocalizations and social behavior with increased aggression, along with comorbidities such as anxiety and motor impairments (Grabrucker et al., 2014, 2016). For the most part, this phenotype was present both in female and male mice. PZD mice were created based on our previous results indicating that Shank2 (SH3 and Multiple Ankyrin Repeat Domains 2) and Shank3 scaffold assembly within the post-synaptic density (PSD) of glutamatergic synapses is zinc-dependent (Baron et al., 2006; Grabrucker et al., 2011a, 2014), and that Shank2 and Shank3 proteins are lost from synapses of PZD animals (Grabrucker et al., 2014). Mutations in *SHANK2* and *SHANK3* are highly associated with monogenetic forms of autism (Grabrucker et al., 2011b; Betancur and Buxbaum, 2013; Guilmatre et al., 2014; Leblond et al., 2014) and PZD mice show behavioral alterations similar to *SHANK* mice modeling ASD by gene knockout.

Zinc is unevenly distributed among brain regions in a hemisphere-specific manner (Wallwork et al., 1984). For example, the hippocampus in the right hemisphere is reported to contain about 20% higher levels of zinc compared to the left hemisphere in rats (Wallwork et al., 1984). Of other minerals tested such as potassium, sodium, magnesium, calcium, iron, copper, and manganese, only zinc and copper displayed this asymmetrical distribution between the right and left hippocampus (Wallwork et al., 1983).

During early brain development, the occurrence of specific patterns in the zinc-containing (zincergic) innervation has been reported. For example, in the striatum of rats, zinc is found enriched in distinct patches at around postnatal day 1 (Vincent and Semba, 1989) that disappear again around day 11. These patches correspond to areas later innervated by dopaminergic neurons. It was speculated that this zincergic innervation serves some early pioneer function, laying down a substrate for later development.

The striatum as part of the basal ganglia receives excitatory inputs from several brain regions such as cortex, thalamic nuclei, hippocampus, and amygdala that form synapses with medium spiny neurons (MSNs) of the dorsal striatum. Both the cortex and the amygdala send zincergic collaterals to the striatum (Sørensen et al., 1995). The dorsal striatum with caudate nucleus, among others, mediates reward value for motivated and intentioned behaviors. These processes were reported influenced by hemispheric differences in striatal dopamine (DA) levels, turnover, and receptor activity (Molochnikov and Cohen, 2014). The reward pathway displays asymmetrical laterality in rodents (Molochnikov and Cohen, 2014).

Interestingly, there is some overlap between ASD symptomatology and striatal function. Therefore, striatal dysfunction is postulated to underlie some characteristic behaviors seen in ASD (Fuccillo, 2016). Imaging studies support this hypothesis as it was shown that the reward circuitry, particularly striatum and ventral prefrontal cortices, displays abnormal activation patterns in ASD (Fuccillo, 2016). Further, in individuals with ASD, the caudate nucleus was reported to show a growth rate twice as high as that of controls independent of overall brain growth (Kohls et al., 2014).

Here, we analyzed brain lateralization in the PZD mouse model on molecular and behavioral level focusing on the striatum, to investigate whether prenatal zinc deficiency leads to an abnormally established lateralization that is maintained after brain development into adulthood. We assessed the expression of marker genes and proteins and performed behavioral tests to evaluate motor lateralization. Moreover, we evaluated lateralization of the striatum using immunohistochemistry and magnetic resonance imaging (MRI).

## Materials and Methods

### Animals

Prenatal zinc deficient mice were generated as described previously (Grabrucker et al., 2014, 2016). In brief, for the generation of PZD animals, 4-week-old C3H/HenRj mice were purchased from Janvier Labs and housed in plastic cages under standard laboratory conditions [22°C, 12 h rhythm (lights on at 7 am)] provided with food and water available *ad libitum*. After 1 week of acclimation, mice were divided into two groups; one group was fed a zinc deficient diet (4 ppm zinc, SSNIFF diets, Germany) with distilled, demineralized drinking water, whereas the control group was fed with standard laboratory food (35 ppm zinc). After 5 weeks, females of the control and zinc deficient group were mated and maintained on their respective diet during pregnancy. All animal experiments were performed in compliance with the guidelines for the welfare of experimental animals issued by the Federal Government of Germany and approved by the Regierungspraesidium Tuebingen and the local ethics committee at Ulm University (ID Number: O.103, 1163, and 1239).

### Chemicals and Reagents

Primary antibodies were purchased from Abcam [protein kinase C (PKCβ), DA receptor D1 (DR1)], Merck Millipore (GAP-43), Novus Biologicals [arrestin beta 2 (ARRB2), GAPDH], Sigma–Aldrich [ACTIN-β, fasciculation and elongation protein zeta 1 (FEZ1)], and Synaptic Systems (BASSOON). Secondary Alexa-coupled antibodies were from Life Technologies. Secondary HRP-coupled antibodies were from Dako. Anti-Mouse IgG + IgM/HRP was obtained from Jackson ImmunoResearch (West Grove, United States). Unless otherwise indicated, all other chemicals were obtained from Sigma–Aldrich.

### Quantitative Real-Time PCR

Isolation of total RNA from eight C3H/HenRj mice per sex and time-point was performed using the RNeasy Plus Universal Midi kit (Qiagen) as described by the manufacturer. Brain hemispheres from four animals of the same sex and age-group were pooled for isolation, except for embryonic day 17 (E17) where all eight hemispheres were pooled. Isolated RNA was eluted in a total of 150 μl RNase-free water and stored at -80°C. First strand synthesis and real-time quantitative real-time PCR (qRT-PCR) amplification were carried out in a one-step, single-tube format using the Rotor-Gene SYBR Green RT-PCR kit (Qiagen) and a Rotor-Gene-Q real-time PCR machine (model 2-Plex HRM) (Qiagen). The qRT-PCR was assayed in RNase free 0.1 ml strip tubes with caps (Qiagen) in a total volume of 20 μl. Amplification conditions were as follows: 10 min at 55°C, 5 min at 95°C, followed by 40 cycles of PCR for 5 s at 95°C for denaturation, 10 s at 60°C for annealing and elongation (one-step). The SYBR Green I reporter dye signal was measured against the internal passive reference dye (ROX). Resulting data were analyzed utilizing the hydroxymethylbilane synthase (HMBS) gene as an internal standard. qRT-PCRs were carried out using validated primer pairs from Qiagen (Quantitect primer assay). Cycle threshold (ct) values were calculated by Rotor-Gene-Q Software (version 2.0.2). All qRT-PCR reactions were run in technical triplicates and mean ct values for each reaction were taken into account for data analysis. Ct values were transformed into virtual mRNA levels according to the formula: virtual mRNA level = 10^{[ct_(target)_-ct_(standard)_]/slope of standard curve}^.

### Protein Biochemistry

Western blot (WB) experiments were performed using S1-fractions of whole brain lysate from one hemisphere. Hemispheres of five control and PZD C3H/HenRj mice were collected in 10 ml HEPES buffer per gram tissue [10 mM HEPES; 0.32 M sucrose, pH 7.42; with protease inhibitor (Roche, Germany)]. The tissue was homogenized using sonication to obtain the CCH-fraction (crude cellular homogenate). To dissociate the nuclear fraction (P1) the CCH-lysate was centrifuged at 3,200 rpm for 15 min at 4°C. Bradford-analysis was performed to measure protein concentration of each sample. A 20 μg of total protein was loaded onto PAGE along with 4× SDS-sample buffer. Evaluation of bands from WBs from three independent experiments was performed using a MicroChemi Imaging System from Biostep and ImageJ. All WB bands were normalized to β-actin or GAPDH.

### Immunohistochemistry

Cryosections were thawed for 20 min and fixed in paraformaldehyde for 20–30 min. After fixation, sections were washed 3× 10 min with PBS followed by permeabilization with 0.2% Triton in PBS for 2 h. The sections were washed 3× 10 min with PBS containing 0.05% Triton and blocked with 10% FCS in PBS for 2 h. Then, sections were incubated with primary antibody at 37°C for 2 h followed by 3× 10 min washing with PBS containing 0.05% Triton. The sections were incubated with the secondary antibody at 37°C for 1.5 h. After washing 3× 15 min with PBS, sections were washed 2× 5 min with PBS containing DAPI and after a final washing step in ddH_2_O, sections were mounted with VectaMount (Vector Laboratories). Images were taken with the same exposure time. The quantification of signal intensities was done using ImageJ 1.51j and AxioVision Software. Background fluorescence was determined, and synaptic fluorescence intensity for all immunoreactive puncta 10% above background was measured from multiple optic fields of view per section.

### Behavioral Analyses

All behavioral experiments were conducted between 9 am and 6 pm. At least 1 h before behavioral testing mice were habituated to the test room. Offspring from nine different mothers was used per group and an equal number of male and female offspring selected per mother. Data from male and female mice are shown pooled as no significant sex-specific effect was detected.

#### Side Preference in a T-Maze Test

C3H/HenRj mice in this experiment were tested at 30 (±3) days of age. The maze (Noldus Information Technology) consisted of two long arms (36.5 cm × 7.5 cm) and one short arm (29.5 cm × 7.5 cm) (start arm), and a center zone (9.5 cm × 9.5 cm) placed in an anechoic chamber. Walls were 12.5 cm in height. Each animal was tested 3× in a row on each day, for five subsequent days. Before testing, animals were habituated for 30 min. For each trial, the animal was placed in the starting arm and given 60 s to decide for one arm (animal entered one arm with all four paws with clear walking direction). If the animal started to turn 180° or more in the middle of the maze before entering an arm, the trial was stopped and a subsequent decision was not counted. After a decision was made or after 60 s, the animal was removed from the maze and placed in a clean cage next to the maze for 30 s. For each trial, the time until the animal left the start arm, a decision for the left arm, the right arm, or absence of decision was recorded. Side preference (SP) of mice is based on the percentage of laterality of a mouse. Percentage of laterality is calculated from the total number of arm decisions per animal. Positive values indicate more decisions to the right; negative values indicate more decision to the left. Animals with >50 and < -50 show a SP.

#### Methamphetamine Induced Rotational Behavior

Methamphetamine (Meth) induced rotation was assessed in C3H/HenRj mice [30 woa (weeks of age)] during a 60 min test session in a plastic cylinder (20 cm × 21 cm). After 10 min habituation mice were injected intraperitoneally (i.p.) with Meth–HCl in 0.9% NaCl solution at a dose of 2.5 mg/kg. Mice were video tracked using EthoVision XT (Noldus, Wageningen, Netherlands). Only full 360° turns were taken as rotation. SP is based on the percentage of laterality of a mouse calculated from the total number of rotations per animal. Positive values indicate more clockwise rotations; negative values indicate more anti-clockwise rotations. Animals with >50 and < -50 show a SP.

All behavioral experiments were conducted in an anechoic chamber under dim red light conditions (15 lux) as preformed by (Rodriguez and Afonso, 1993). The experimental set up was cleaned with 70% ethanol between each trial in order to eliminate odor traces between animals.

### Magnetic Resonance Imaging

High-resolution MRI experiments on age- and sex-matched control and PZD mice were carried out under isoflurane anesthesia (5% for induction, 1.5% for maintenance, mixed with air). PZD mice were generated as described previously (Grabrucker et al., 2014, 2016) using C57BL/6 mice purchased from Janvier Labs. All data were acquired on a dedicated small bore animal scanner (Biospec 117/16, Bruker, Ettlingen, Germany) equipped with a cryogenically cooled two-element surface (MRI CryoProbe^TM^, Bruker BioSpec, Ettlingen, Germany) transmit/receive coil. Anatomical brain images were acquired in axial, sagittal, and coronal slice orientation applying a gradient-echo (FLASH) sequence with acquisition parameters as: TE/TR 2.2/193 ms, matrix 260 × 260, Δ*r* = 65 × 65 × 500 μm^3^). Analysis followed procedures published in Wiesner et al. (2015).

### Statistics

Statistical analysis was performed with GraphPad Prism 7. Data are shown as mean ± SEM. Significances are stated with *p*-values <0.05^∗^; <0.01^∗∗^; <0.001^∗∗∗^.

## Results

### Marker Genes and Proteins for Brain Lateralization Are Altered in PZD Mice after Birth

**FIGURE 1:**
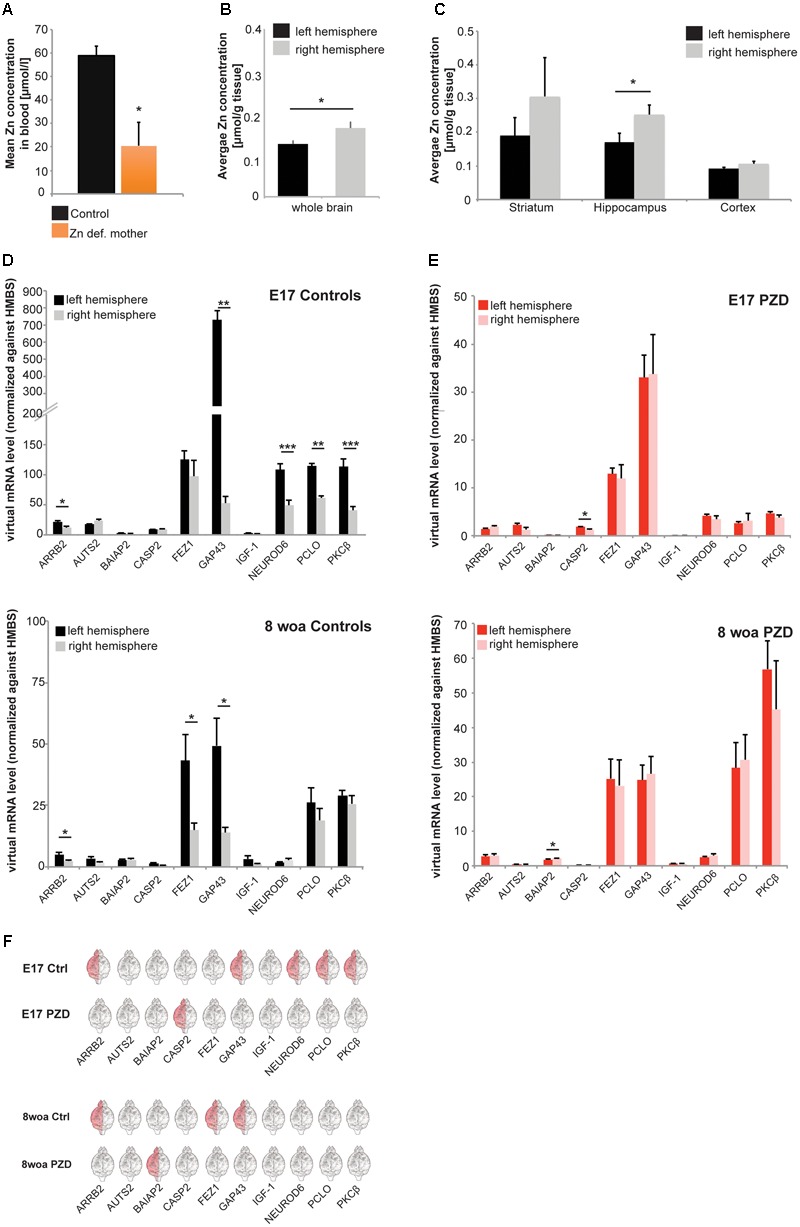
Determination of brain region and hemisphere-specific zinc levels in the mouse brain and analysis of marker genes for brain lateralization in PZD mice. **(A)** Whole-blood Zn levels of mothers measured by AAS in three animals per group. Animals on a zinc deficient diet show significantly reduced zinc levels compared to mice on the control diet. **(B,C)** Zinc concentrations of right and left hemisphere were determined by AAS in mice at 8 weeks of age (woa, *n* = 10). **(B)** Significantly higher zinc levels can be found in the right hemisphere (*t*-test, *p* = 0.017). **(C)** A brain region-specific analysis shows higher levels of zinc in the right hemisphere in striatum and cortex (non-significant), and hippocampus (significant, *t*-test, *p* = 0.049). **(D–F)** mRNA was isolated from both hemispheres and selected marker genes were analyzed for expression levels at different developmental time-points. All values were normalized against HMBS expression. For the analysis, eight animals per group were used and analyses performed in technical triplicates. Statistical analysis was performed using *t*-test. **(D)** Control animals show a hemisphere-specific expression of ARRB2 (significantly higher expression in the left hemisphere), GAP43 (significantly higher expression in the left hemisphere), NEUROD6 (significantly higher expression in the left hemisphere), PCLO (significantly higher expression in the left hemisphere), and PKCβ (significantly higher expression in the left hemisphere), at embryonic day 17 (E17). At 8 woa, control mice maintain a hemisphere-specific expression of ARRB2 (significantly higher expression in the left hemisphere), FEZ1 (significantly higher expression in the left hemisphere), and GAP43 (significantly higher expression in the left hemisphere). **(E)** In PZD mice, only CASP2 showed hemisphere-specific expression at E17 and at 8 woa BAIAP2 (significantly higher expression in the right hemisphere). **(F)** Direct comparison of lateralization of gene expression of marker genes. In control animals, higher expression is mostly found in the left hemisphere for most genes. Differences between hemispheres of genes expressed lateralized in controls are absent in PZD mice.

To generate PZD mice, we induced a mild maternal zinc deficiency throughout pregnancy as performed previously for the studies reporting ASD-like behavior in PZD mice (Grabrucker et al., 2014, 2016). The dietary zinc deprivation lead to a significant reduction in blood zinc levels in mothers fed the zinc deficient diet during pregnancy (**Figure 1A**). It was shown before that a similar and significant reduction of maternal blood zinc levels caused a reduction in brain zinc levels in pups (Grabrucker et al., 2014, 2016).

Further, we wanted to determine whether the previously reported hemisphere-specific distribution of zinc in rats (Wallwork et al., 1984) is also present in the wild type mice used in this study. To that end, the brains were prepared from 10 male mice at 8 woa and zinc concentrations determined by atomic absorption spectrometry (AAS) (**Figures 1B,C**). The results confirm a significantly higher zinc concentration in the right hemisphere compared to the left hemisphere (**Figure 1B**). As reported before for rat, the hippocampus showed the most obvious difference between the hemispheres, with a significantly higher level of zinc on the right side (**Figure 1C**). Thus, we investigated whether this unequal distribution of zinc is not only a result of hemisphere dominance but involved in the establishment of brain region-specific lateralization between brain hemispheres.

Therefore, we set out to determine useful biochemical markers for brain lateralization in mice. To that end, we selected 10 different genes derived from a list of over 500 genes compiled from multiple studies on humans, wild type mice and rats (Guarneri et al., 1988; Larisch and Klimke, 1988; Sun et al., 2005; Moskal et al., 2006; Sun and Walsh, 2006; Orman and Stewart, 2007; Ribasés et al., 2009; Samara et al., 2011). The selection was based on different parameters: (i) frequency of gene mentioned in publications on asymmetric expression, (ii) asymmetrical expression overlaps in human and rodents, (iii) expression differences reported across several developmental time-points, (iv) expression differences detected on both, protein and mRNA level. Based on these parameters, a ranking of candidate genes was established and the following genes further analyzed as putative marker genes: ARRB2, AUTS2 (autism susceptibility candidate 2), BAIAP2 (BAI1-associated protein 2), CASP2 (caspase 2), FEZ1, GAP43 (growth associated protein 43), IGF1 (insulin-like growth factor 1), NEUROD6 (neuronal differentiation 6), PCLO1 (piccolo 1), and PKCβ. The marker CASP2 was included, because some caspase-family members show asymmetrical expression and because there is evidence for an altered expression of many caspase family members in ASD patients (Siniscalco et al., 2012).

We prepared brain lysate of two different developmental time-points (E17 and 8 woa) from both sexes and from the left and right hemisphere of control mice and analyzed the expression of these genes between the two hemispheres by qRT-PCR (**Figures 1D–F**). The results show that indeed, significant differences in the expression of most marker genes comparing left and right hemispheres can be detected during embryonic brain development at E17 (**Figures 1D** upper panel,**F**). Control animals show a hemisphere-specific expression of ARRB2, GAP43, NEUROD6, PCLO, and PKCβ. Some of the marker genes show ongoing lateralized expression during aging. At 8 woa, control mice still show a hemisphere-specific expression of ARRB2, FEZ1, and GAP43 (**Figures 1D** lower panel,**F**). Among the genes that show lateralized expression in control mice, none was detected with similar expression patterns in PZD animals. We could not detect hemisphere-specific expression for the selected genes in PZD mice at E17 and 8 woa (**Figures 1E,F**). However, CASP2 (at E17) and BAIAP2 (at 8 woa) showed lateralized expression that was not present in control mice. No differences between male and female mice were detected (data not shown) and mice of both sexes pooled.

**FIGURE 2:**
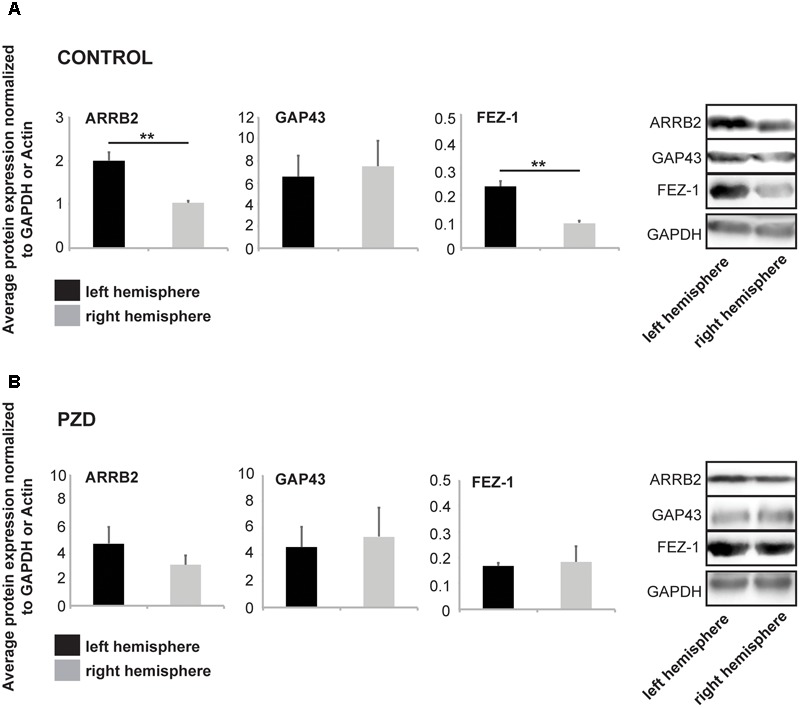
Marker proteins for brain lateralization are altered in PZD mice. Protein was isolated from hippocampi of both hemispheres and selected marker proteins were analyzed for expression levels at 8 woa. All values were normalized against GAPDH or β-Actin expression. For the analysis, three male animals per group were used and analyses performed in technical triplicates. Statistical analysis was performed using *t*-test. **(A)** In control mice, similar to the expression on mRNA level, ARRB2 and FEZ-1 are significantly higher expressed on protein level in the left hemisphere (ARRB2 *p* = 0.0058; FEZ-1 *p* = 0.0042). **(B)** Analysis of the same markers in PZD mice reveals no significant differences in the concentration of ARRB2, GAP43, or FEZ-1 between left and right hemisphere.

Based on these results, we next investigated whether the observed differences between control and PZD mice are also visible on protein level. We selected the marker genes that showed lateralized expression throughout development (E17) and aging (8 woa) (ARRB2, GAP43, and FEZ1) and investigated hippocampal and striatal lysates from three animals at 8 woa using WB analysis (**Figures 2A,B** and **Supplementary Figure S1**). The results show that control mice also show lateralized expression of ARRB2 and FEZ-1 in the hippocampus on protein level. Similar to the mRNA levels, the expression was significantly higher in the left hemisphere (**Figure 2A**). In PZD animals, in contrast, no significant differences were detected between the left and right hemisphere (**Figure 2B**). In the striatum, control and PZD mice show no lateralization of the marker proteins, but PZD mice show less expression of total FEZ1 (**Supplementary Figures S1A,B**).

### PZD Mice Show Altered Behavior in Tests for Motor Lateralization

Based on the biochemical alterations observed in the previous experiments, we next performed behavioral assays to investigate whether the altered gene or protein expression is accompanied by a change in functional hemispheric dominance. To that end, mice were first subjected to tests determining a general motor lateralization by SP in a T-maze test. PZD mice developed normally after birth and no weight differences were detected at the time of testing.

**FIGURE 3:**
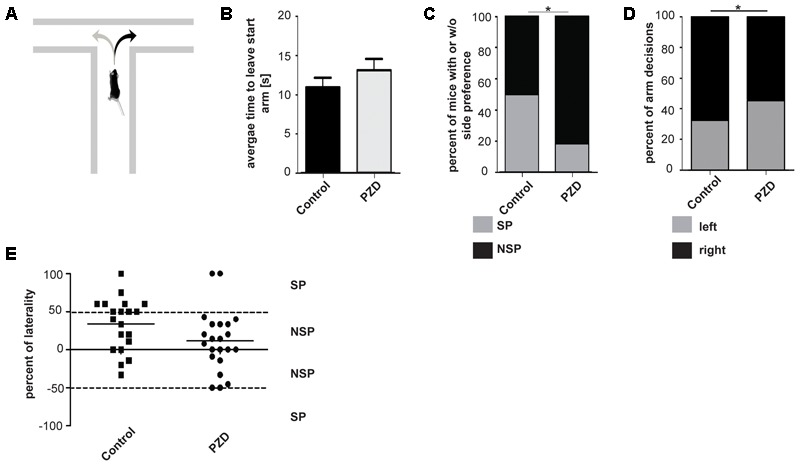
Side preference in the T-maze of PZD mice vs. controls. **(A)** Mice were introduced to a T-maze and side preference measured. **(B)** Bar graph representation of the mean time to leave the start arm of each group [PZD animals, *n* = 22 (11 females and 11 males); control animals, *n* = 20 (10 females and 10 males)]. No significant differences could be observed (significance levels were calculated using the unpaired Student’s *t*-test). **(C)** Bar graph representation of the percentage of animals with side preference (SP) and with no side preference (NSP) shown in **(E)**. Number of animals: PZD, *n* = 22; control, *n* = 20. Significance levels were calculated from absolute numbers using Fisher’s exact test. A significant difference could be observed (*p* = 0.0488). **(D)** Bar graph representation of percent of right and left turns per group from individuals shown in **(E)**. A significant difference could be observed (*p* = 0.026). **(E)** Scatter dot blot representation of the individual percentage of laterality of PZD and control animals in the T-maze. Percentage of laterality is calculated from the total number of arm decisions per animal. Positive values indicate more decisions to the right; negative values indicate more decision to the left. The mean of each group is shown as horizontal bar. Dashed lines indicate the threshold of animals with and without side preference. Total number of decisions: 167 from 22 PZD animals; 137 from 20 control animals.

The T-maze test is a task to assess working memory (cognitive functions) in rodents, but can also be used to analyze SP of motor functions (Rodriguez et al., 1992; Deacon and Rawlins, 2006). An innate SP in the T-maze was shown in rodents on the individual as well as on population-level (Castellano et al., 1987; Andrade et al., 2001) (**Figure 3A**). In the T-maze used in this study, no aversive or appetitive stimuli were used to motivate the animals to leave the start arm and to make a decision between the left and right arm, and we could not observe significant differences between the groups in the mean time to leave the starting arm (**Figure 3B**). We analyzed SP in control and PZD mice and found that the percentage of animals showing SP was significantly higher in control animals compared to PZD mice (**Figures 3C,E**). Further, a preference for turns to the right found in control mice was significantly less pronounced in PZD animals (**Figures 3D,E**). We conclude that control mice show a right-side preference in the T-maze test and that PZD mice results in a loss of this preference.

Given that it was reported that turning behavior in a maze may be dependent on a functional striatum (Estrada-Sánchez et al., 2013) and that turning behavior can even be induced by manipulation of striatal activity (Souilhac et al., 1995), we next performed a test focusing on lateralization of the striatum. Further, the striatum was reported to be a brain region affected in ASD, and ASD mouse models such as Shank3 mutant mice display striatal dysfunction (Peça et al., 2011; Jaramillo et al., 2016; Wang et al., 2016).

**FIGURE 4:**
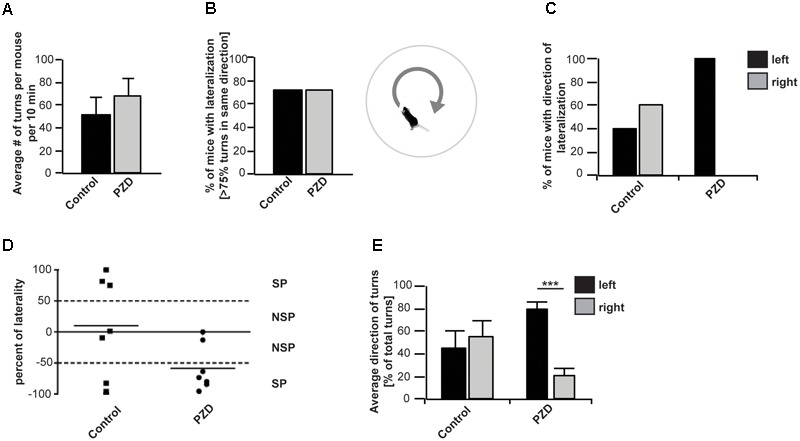
Orientation preference of PZD and control mice. Mice received an i.p. injection of methamphetamine and were placed in the center of a round arena. Their behavior was recorded for 60 min and the number of left- and rightward rotations measured. **(A)** No significant difference between control mice and PZD mice can be observed regarding the average number of rotations (full 360° turns with no longer than 2 s pause of movement within one rotation) per mouse. **(B)** The direction of each rotation was detected and in case of more than 75% of rotations in one direction, the mouse was counted as mouse with lateralized rotational behavior. In both groups, 71% of the animals tested showed a preference for rotations in a certain direction. **(C)** A total of 40% of control mice with lateralized rotational behavior preferred to turn anti-clockwise (left), and 60% clockwise (right). All (100%) of PZD mice with lateralized rotational behavior showed preference for the anti-clockwise direction. **(D)** Scatter dot blot representation of the individual percentage of laterality of PZD and control animals. Percentage of laterality is calculated from the total number of rotations per animal. Positive values indicate more clockwise rotations; negative values indicate more anti-clockwise rotations. The mean of each group is shown as horizontal bar. **(E)** The average percentage of left and right turns from the total number of turns was measured for each mouse and the average of all mice per group is shown. On average, control mice performed similar amounts of left and right turns. PZD mice turn significantly more often anti-clockwise than clockwise. Comparison of the average percentage of left and right turns from the total number of turns between control and PZD mice shows more left turns and less right turns in PZD mice (one-way ANOVA *F*_3,24_ = 4.304; *p* = 0.015, post-test, PZD_left_ vs. PZD_right_ *p* < 0.001) [PZD animals, *n* = 7; control animals, *n* = 7 (four females and three males each)].

Several studies could show a rotational preference of rats dependent on the level of DA within the right of left striatum (Glick and Cox, 1978; Lindemann et al., 2001). As behavioral index for mesostriatal DA activity we used the paradigm of methamphetamine induced rotational behavior in non-lesioned mice. When methamphetamine is administered to mice, they turn in circles in one dominant direction. This behavioral asymmetry is thought to be due to an endogenous asymmetry of the mesostriatal DA system (Jerussi and Glick, 1974; Glick and Cox, 1978; Bracha et al., 1987; Lindemann et al., 2001) (**Figures 4A–E**). Our results show that on average, control mice and PZD mice performed a similar number of rotations within the tested time period (**Figure 4A**). In both groups, 70% of the animals tested showed a preference for rotations in a certain direction. The preference was stated in case more than 75% of all turns of one animal during the test session were in a particular direction (**Figure 4B**). While control mice either preferred to turn anti-clockwise (left, 40%) or clockwise (right, 60%), all of the PZD mice tested that showed preference for one direction and turned anti-clockwise (left, 100%) (**Figures 4C,D**). This is also reflected in the average percentage of left and right turns from the number of total turns across all tested mice (**Figure 4E**). While, averaging all mice per group, control mice performed similar amounts of left and right turns, PZD mice turn significantly more often anti-clockwise than clockwise.

In mice, asymmetric orientation behavior is based on the asymmetry of activity between the left and right basal ganglia including the striatum, or the left or right frontal cortex (Bracha et al., 1987). Studies have shown that mice rotate toward the hemisphere that has less striatal dopaminergic activity (Jerussi and Glick, 1974). Thus, the directional preference of mice can provide information on the endogenous asymmetry of the striatal DA system (Jerussi and Glick, 1974; Brundin et al., 1986). Our results show a strong leftward orientation of PZD mice, indicating abnormal lateralization with a stronger activation of the right striatum.

### PZD Mice Show Altered Functional Striatal Lateralization and Morphology

**FIGURE 5:**
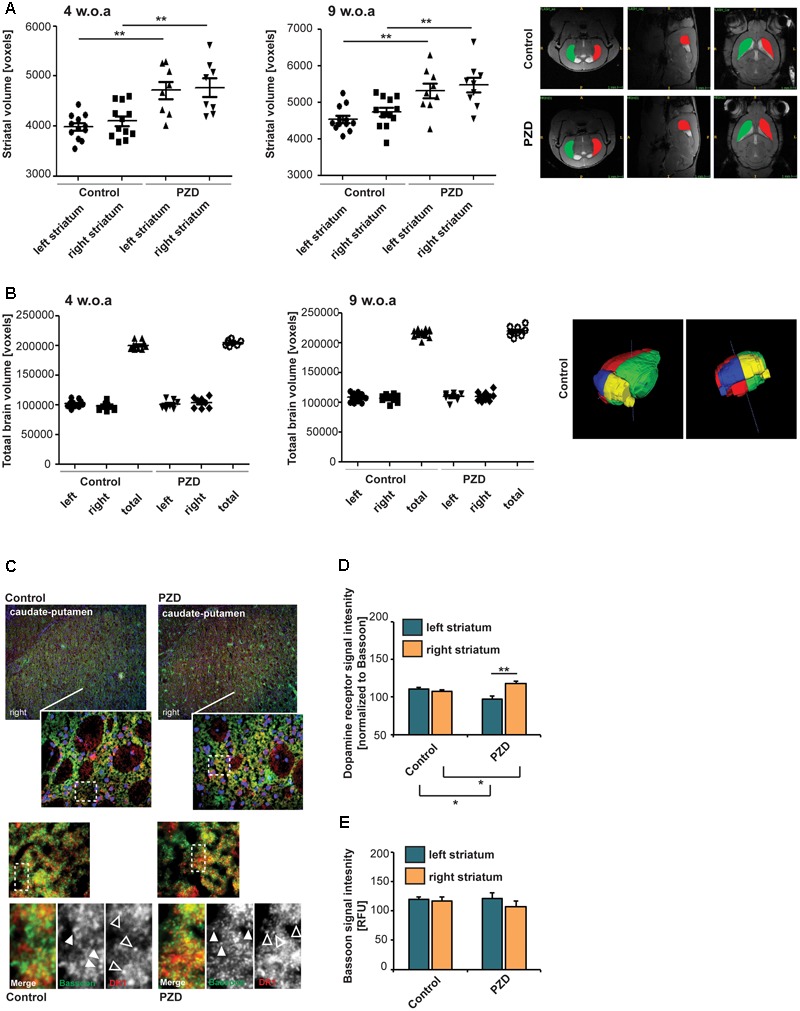
Prenatal zinc deficient mice show altered dopamine receptor 1 (DR1) lateralization and striatal morphology. **(A)** Volumetric measurements of striatal volume of mice at postnatal day (PD) 29 (left panel) and PD64 (right panel). Volumes of the striatum were determined per individual from left and right hemisphere. Compared to controls, PZD mice show an enlarged striatum that is maintained into adulthood (two-way ANOVA with Bonferroni *post hoc* test: PD29, control_left_ vs. PZD_left_: *p* = 0.001; control_right_ vs. PZD_right_: *p* = 0.004; PD64, control_left_ vs. PZD_left_: *p* = 0.004; control_right_ vs. PZD_right_: *p* = 0.008; *n* = 8 PZD (four females and four males), *n* = 12 control (six females and six males)]. Exemplary sections of control and PZD in all dimensions are depicted with colored areas indicating the left and right striatum. **(B)** The total volume of the brain hemispheres and thus total brain volume was not significantly different between PZD and controls. A 3D-rendered image of a control brain at PD64 from two different perspectives is shown. **(C)** Coronal sections from three control and three PZD mice were stained for the pre-synaptic marker Bassoon and DR1 (merged images show additional DAPI staining of nuclei). The signal intensity of immunoreactive puncta was analyzed in four optic fields of view in three consecutive sections per mouse within the left and right striatum. **(C,D)** DR1 signal intensities (open arrows) were normalized to Bassoon (closed arrows). **(D)** A significant difference was detected between the left and right striatum of PZD mice (one-way ANOVA *F*_3,8_ = 9.684; *p* = 0.005, Bonferroni post-test, PZD_left_ vs. PZD_right_ *p* = 0.0092). Compared to controls, the DR1 expression was increased in the right striatum in PZD mice (*p* = 0.0366) and decreased in the left striatum (*p* = 0.0428). **(E)** The BASSOON signal intensities were not different between control and PZD mice and between hemispheres (one-way ANOVA *F*_3,8_ = 0.634; *p* = 0.613).

Studies have shown that the left nucleus caudatus, a component of the striatum, has a larger volume compared to the right in healthy people (Zabó et al., 2003). In autistic patients, however, a larger volume of the right nucleus caudatus was observed compared to the control group along with a larger volume of the striatum (caudate + putamen) (Hollander et al., 2005; Haznedar et al., 2006). Therefore, we wanted to investigate whether PZD mice show alterations in striatal volume. To this end, we performed small animal MRI at two developmental time-points. Although we could not detect a hemisphere-specific difference in striatal volume, in line with the observation in humans with ASD (Hollander et al., 2005), volumetric measurements of total striatal volume in juvenile mice showed an increase in PZD mice compared to controls that is maintained in adult mice (**Figure 5A**). We found no evidence for structural lateralization in the striatum in control mice. The total brain size of PZD mice was not significantly different in comparison to controls (**Figure 5B**).

A right–left asymmetry of the striatum may also be detected biochemically by an asymmetric distribution of DA receptors (Larisch and Klimke, 1988; Larisch et al., 1998). Thus, to further analyze the striatum of PZD mice, we performed immunohistochemistry to quantify the expression of DA receptors. To that end, we stained coronal sections from control and PZD mice for the pre-synaptic marker BASSOON and DR1 (**Figures 5C,D**). The signal intensity of immunoreactive puncta was measured. While we could not detect a significant difference in synaptic BASSOON expression levels between controls and PZD mice and between the left and right hemisphere (**Figures 5C,E**), we detected a significant difference in DR1 levels between the left and right striatum of PZD mice (**Figure 5D**). Compared to controls, the DR1 expression was increased in the right striatum in PZD mice, and decreased in the left striatum. DR1 are located on the MSNs of the direct striatonigral pathway and DR1 agonists were shown effective in causing rotational behavior in unilateral striatonigral lesioned rats (Ruskin et al., 1999). The direct pathway ultimately influences basal ganglia output nuclei, which in turn regulate rotational behavior via thalamocortical and brainstem motor circuits. Thus, increased expression levels of DR1 in the right hemisphere are in line with leftward rotation-behavior in PZD mice.

## Discussion

Asymmetric behavioral functions in animals and humans include mainly auditory and sensory perception and motor preferences (Toga and Thompson, 2003). A disruption of normal brain asymmetry can be found in different neurological disorders, including psychiatric disorders like ASD. Anatomically, these disruptions can be seen as a reduction or even reversal of asymmetric structures (Sun and Walsh, 2006) but also as specific behavioral impairments in human patients with ASD.

Zinc is one of the most prevalent trace metals in the brain and acts as neuromodulator or neurotransmitter at excitatory synapses within glutamatergic pathways (Slomianka, 1992; Brown and Dyck, 2004; Bitanihirwe and Cunningham, 2009). Glutamatergic neurotransmission is especially in focus of research regarding possible pathomechanisms of ASD (Yang and Chang, 2014). Zinc deficiency is implicated in several neurological disorders (Pfaender and Grabrucker, 2014). In particular, a role of prenatal zinc deficiency was proposed in the etiology of ASD (Yasuda et al., 2011; Grabrucker, 2012; Arora et al., 2017).

Zinc is one of the very few trace elements showing unequal distribution between specific regions of the right and the left hemisphere (Valdes et al., 1982; Wallwork et al., 1983). Here, we confirmed the unequal distribution of zinc in the brain of mice. It is possible that the distribution of zinc is not only consequence of lateralization but also exerts modulatory effects on lateralization by promoting region-specific synapse formation and maintenance in one hemisphere. As synaptic vesicles loaded with zinc often appear in later developmental stages (Valente and Auladell, 2002; Valente et al., 2002), the role of zinc as post-synaptic component might be contributing to these processes. At the PSD zinc associates with the scaffold proteins Shank2 and Shank3. Not only have mutations in these proteins been associated with ASD in humans and ASD-like behavior in mice similar to PZD mice, but Shank3 mice also show aberrant structural connectivity and striatal defects (Wang et al., 2016).

Here, we analyzed PZD mice regarding brain lateralization. In different studies, genomic screening approaches have been used to identify genes that are differentially expressed between hemispheres. For example, analysis of fetal human brains for differentially expressed genes revealed several genes with consistently lateralized expression (Sun et al., 2005). It was concluded that the asymmetry of the human cortex is accompanied by an early transcriptional asymmetry. Among the identified genes were the markers ARRB2, AUTS2, BAIAP2, FEZ1, GAP43, IGF1, and NEUROD6. Most of those markers were verified in a study on rat hippocampal lateralization markers (Moskal et al., 2006). As these genes have previously been associated with the development of psychiatric diseases such as ASD, they were included in this study. Although, loss of ARRB2 in mice causes alterations in both the dopaminergic and opioid system (Björk et al., 2013), and loss of FEZ1 causes hyperactivity and enhanced mesolimbic dopaminergic transmission (Sakae et al., 2008), their role in brain lateralization has not been investigated so far. In general, lateralization of the observed markers was absent in PZD animals on mRNA and protein level indicating abnormal brain asymmetry in these animals. As these alterations already occur in the embryonic phase, it is unlikely that they are a consequence of the behavioral differences seen in PZD mice. Prenatal zinc deficiency may affect neuronal connectivity during brain development which will feed back into differences in brain region activity and activated signaling pathways and lead to altered expression of many genes.

On behavioral level, lateral biases are widespread also among non-human vertebrates (Vallortigara and Rogers, 2005). For example, motor functions are lateralized in mice (Collins, 1975). Thus, we focused on lateralization of motor functions in two different setups where lateralization has been reported for rodents before. We found significant differences in lateralization between PZD animals and controls in the T-maze test. A rightward bias in spontaneous arm preference has been described before for wild type animals (Castellano et al., 1987; Andrade et al., 2001). In line with these studies, tested control mice showed a rightward bias. PZD mice displayed a reduction in SP, and in case they favored one side, there was no bias toward right turns. As the degree of SP was reported to decrease in the T-maze during postnatal development in rats (Rodriguez and Afonso, 1993; Andrade et al., 2001), testing at earlier time-points might have revealed an even more severe phenotype.

Altered turning behavior may hint toward abnormal lateralization of the striatum. Thus, we elicited rotational behavior through activation of striatal pathways by injection of methamphetamine and investigated differences between PZD and control mice. It has been shown before that rats tested for amphetamine-induced rotation had right side biases (54.8%) (Jerussi and Glick, 1974). This result has been obtained using 602 rats. In our study, using seven mice, we detected a slight trend toward more rightward rotations in control animals that, however, was statistically not significant. The right side bias of rodents in the T-maze may be stronger and was also detected in previous studies with a lower amount of experimental animals.

Interestingly, we could show a SP in rotational behavior in PZD mice. In contrast to the expected rightward bias of control mice, PZD mice showed a significant leftward bias that can be associated with increased activation of the right striatum. The presence of SP, although with side reversal, in PZD mice in the test for rotational behavior is not inconsistent with the loss of SP in the T-maze test as the behavior of mice in both tests is under the influence of several different brain regions. For example, spatial preference as tested in the T-maze paradigm has been shown to also depend on dopaminergic neurotransmission in the hippocampus (Skirzewski et al., 2017). Differences in hippocampal zinc levels between the right and left hemisphere have been correlated with spatial preference of rats before (Valdes et al., 1982).

In line with the behavioral phenotype, we detected significantly higher DR1 levels in the right caudate-putamen of PZD mice. In human ASD patients, the volume of the striatum (caudate + putamen) was found increased with the right caudate volume found significantly larger in autistic subjects than in controls. Right caudate and total putamen volumes correlated positively with repetitive behavior scores (Hollander et al., 2005). Using brain imaging, we also detected an abnormal total striatal volume in PZD mice along with no increase in total hemisphere volume and total brain volume. As we did not evaluate the caudate and putamen separately due to the size limitations, it may be possible that a specific increase in the right caudate is masked by the overall increase in striatal size in PZD mice. The striatal volume was increased in both young and adult PZD mice with slightly less increased size in adult mice, which may be associated with the reported decrease in SP in the T-maze test (Rodriguez and Afonso, 1993; Andrade et al., 2001).

The increased functional lateralization of the striatum is not contradictory to a loss of lateralized gene and protein levels detected in whole brain samples. The striatum receives input from the cortex, thalamus, and the direct striatonigral pathway and projects into specific downstream pathways playing an essential role in movement control via the basal ganglia. Abnormal connectivity and lateralization of input areas may result in altered, and possibly increased lateralization of the striatum. Further, loss of lateralization in brains of human ASD patients is reported also along with increased lateralization of the striatum. Interestingly, it was proposed that the striatum might occupy a central place in ASD pathophysiology (Fuccillo, 2016).

Taken together, the results obtained in this study propose a novel role for zinc signaling in the establishment of brain lateralization during development and suggest that PZD animals display a disturbed lateralization of brain functions, which may lie at the core of ASD-like behavior.

## Author Contributions

JCH and NK performed behavioral and molecular analyses, and edited the manuscript. AKS, HA, AA, and MS carried out the MRI experiments, and revised the manuscript. VR and TMB helped in organizing the structure of the work, setting the experimental procedures, and edited the manuscript. AMG and SG conceived the study, participated in its design, coordination and data analysis, and drafted the manuscript. All authors read and approved the final manuscript.

## Conflict of Interest Statement

The authors declare that the research was conducted in the absence of any commercial or financial relationships that could be construed as a potential conflict of interest.

## Supplementary Material

FIGURE S1 Marker proteins for brain lateralization in PZD mice. Protein was isolated from striatum of both hemispheres and selected marker proteins were analyzed for expression levels at 8 woa. All values were normalized against GAPDH expression. For the analysis, three male animals per group were used and analyses performed in technical triplicates. Statistical analysis was performed using *t*-test. **(A)** In control mice, ARRB2 and FEZ-1 are not significantly higher expressed on protein level in the left hemisphere (FEZ-1 *p* = 0.0603). **(B)** Analysis of the same markers in PZD mice reveals no significant differences to control mice, although total FEZ1 levels are significantly less in PZD mice compared to controls (left hemisphere FEZ1 control vs. PZD, *p* = 0.0016; right hemisphere FEZ1 control vs. PZD, *p* = 0.0043).
