# Supplementary figures and images for: Brain Lateralization in Mice Is Associated with Zinc Signaling and Altered in Prenatal Zinc Deficient Mice That Display Features of Autism Spectrum Disorder
Source: Front Mol Neurosci. 2018 Jan 15;10:450. doi: 10.3389/fnmol.2017.00450 (PMC5775238; doi:10.3389/fnmol.2017.00450)

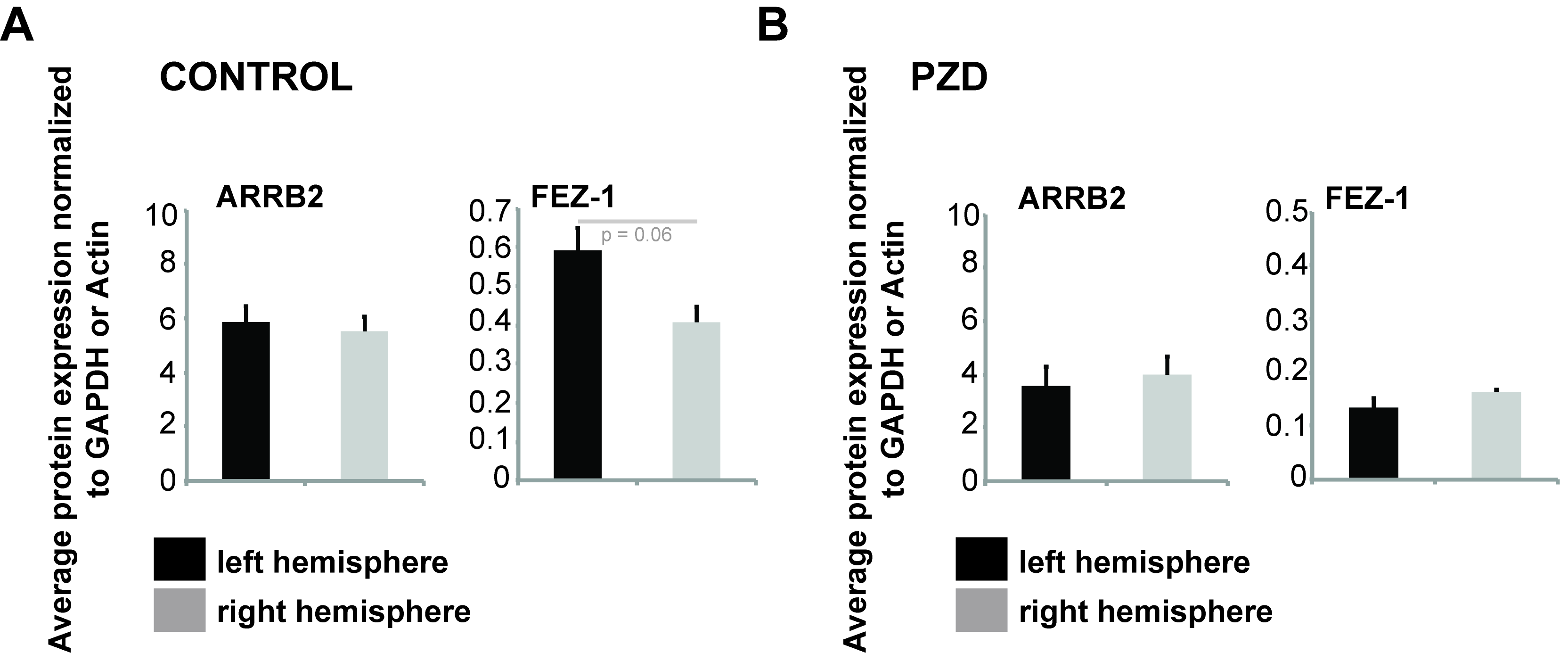

Supplement: FIGURE S1 — Marker proteins for brain lateralization in PZD mice. Protein was isolated from striatum of both hemispheres and selected marker proteins were analyzed for expression levels at 8 woa. All values were normalized against GAPDH expression. For the analysis, three male animals per group were used and analyses performed in technical triplicates. Statistical analysis was performed using t-test. (A) In control mice, ARRB2 and FEZ-1 are not significantly higher expressed on protein level in the left hemisphere (FEZ-1 p = 0.0603). (B) Analysis of the same markers in PZD mice reveals no significant differences to control mice, although total FEZ1 levels are significantly less in PZD mice compared to controls (left hemisphere FEZ1 control vs. PZD, p = 0.0016; right hemisphere FEZ1 control vs. PZD, p = 0.0043). [file Image_1.TIF]
